# Citrus rootstocks modify scion antioxidant system under drought and heat stress combination

**DOI:** 10.1007/s00299-021-02744-y

**Published:** 2021-07-07

**Authors:** Damián Balfagón, Fátima Terán, Tadeu dos Reis de Oliveira, Claudete Santa-Catarina, Aurelio Gómez-Cadenas

**Affiliations:** 1 Departamento de Ciencias Agrarias y del Medio Natural, Universitat Jaume I, 12071 Castellón de la Plana, Spain; 2 Centro de Biociências e Biotecnologia (CBB), Laboratório de Biologia Celular e Tecidual (LBCT), Universidade Estadual do Norte Fluminense Darcy Ribeiro (UENF), Av. Alberto Lamego 2000, Campos dos Goytacazes, RJ 28013-602 Brazil

**Keywords:** Abiotic stress, Climate change, Grafting, H_2_O_2_, Stress combination

## Abstract

**Key message:**

The activation of the antioxidant system under stress combination is a transmissible trait from the rootstock to the scion. Therefore, rootstock selection is key to improve crop performance and a sustainable production under changing climate conditions.

**Abstract:**

Climate change is altering weather conditions such as mean temperatures and precipitation patterns. Rising temperatures, especially in certain regions, accelerates soil water depletion and increases drought risk, which affects agriculture yield. Previously, our research demonstrated that the citrus rootstock Carrizo citrange (*Citrus sinensis* × *Poncirus trifoliata*) is more tolerant than Cleopatra mandarin (*C. reshni*) to drought and heat stress combination, in part, due to a higher activation of the antioxidant system that alleviated damage produced by oxidative stress. Here, by using reciprocal grafts of both genotypes, we studied the importance of the rootstock on scion performance and antioxidant response under this stress combination. Carrizo rootstock, under stress combination, positively influenced Cleopatra scion by reducing H_2_O_2_ accumulation, increasing superoxide dismutase (SOD) and ascorbate peroxidase (APX) enzymatic activities and inducing SOD1, APX2 and catalase (CAT) protein accumulations. On the contrary, Cleopatra rootstock induced decreases in APX2 expression, CAT activity and SOD1, APX2 and CAT contents on Carrizo scion. Taken together, our findings indicate that the activation of the antioxidant system under stress combination is a transmissible trait from the rootstock to the scion and highlight the importance of the rootstock selection to improve crop performance and maintain citrus yield under the current scenario of climate change.

**Supplementary Information:**

The online version contains supplementary material available at 10.1007/s00299-021-02744-y.

## Introduction

Wild and cultivated plants are affected by environmental changes that are detrimental to their development and can reduce agriculture production. Among them, drought and high temperatures are important stress factors for agriculture areas worldwide (Li et al. 2009; Zandalinas et al. 2018). Water deficit causes stomatal closure to prevent water loss by transpiration; subsequently, CO_2_ intake is reduced impairing photosynthesis, which, in turn, unbalances electron transport chain and causes oxidative stress (Pinheiro and Chaves 2011; Vincent et al. 2020). Heat stress increases plant transpiration and photosynthesis rates, alters cell membrane structure and causes protein denaturation and enzyme inactivation (Wahid et al. 2007). Both stress conditions have a strong impact on plant growth, development and productivity. In addition, the probability that drought and heat stress impact simultaneously on plants is increasing due to climate change (Zhao et al. 2017; Zandalinas et al. 2018; Teuling 2018). Recent studies have demonstrated that plant responses to stress combination are unique and not only the addition of responses to individual stresses (Zandalinas et al. 2018, 2019; Balfagón et al. 2019). Specifically, the impact of drought and heat stress combination on plants is more damaging than the effect caused by the individual stresses (Rizhsky et al. 2002, 2004; Fahad et al. 2017; Zandalinas et al. 2018; Elferjani and Soolanayakanahally 2018; Fábián et al. 2019; Balfagón et al. 2020).

Grafting is an ancient and widespread agronomical practice. By the use of a rootstock, it is possible to modify the phenotype of commercial varieties that constitute plant aerial part. This technique implies several advantages, such as resistance to biotic and abiotic stress factors or increased crop production (Albacete et al. 2015). Through hydraulic and molecular signals, the rootstock can modify scion physiology and genetic expression (Marguerit et al. 2012; Cookson and Ollat 2013; Albacete et al. 2015; Gautier et al. 2019). Therefore, grafting is commonly used in agriculture to confer to productive varieties profitable traits. *Momordica charantia* was used as a rootstock to transfer thermotolerance traits to *Cucumis sativus* scions by increasing the expression of key enzymes and improving photosynthesis in the scion (Xu et al. 2018). Moya et al. (2002), showed in citrus that salt tolerance traits such as increased water use efficiency and reduced leaf Cl^−^ accumulation were transmitted from the tolerant rootstock to the scion. In modern citrus, industry grafting is always used and elite productive varieties are grafted onto resistant rootstocks to improve yield and tolerance to abiotic and biotic stress factors (Bowman and Joubert 2020).

At low levels, reactive oxygen species (ROS), which are normally present in aerobic cells, are considered signaling transduction molecules (Mittler 2016). However, ROS overaccumulation in cells or organelles, caused by metabolic imbalances due to environmental stresses, leads to oxidative stress and cell damage (Suzuki et al. 2012). Cell antioxidant defense system, what encompasses antioxidant enzymes and molecules, is crucial in detoxifying ROS to avoid excessive cell death and massive plant damage (Mittler 2002; Hossain et al. 2009; Foyer and Shigeoka 2011). In Zandalinas et al. (2017), it was shown that Carrizo citrange had higher tolerance to stress combination (drought and heat) than Cleopatra mandarin due to the accumulation of soluble antioxidant compounds and the increase of superoxide dismutase (SOD), catalase (CAT) and APX enzyme activities.

In the present work, we hypothesize that activation of the antioxidant system under drought and heat stress combination is a transmissible trait from the rootstock to the scion. Using reciprocal grafts between Carrizo and Cleopatra genotypes, we prove this hypothesis showing both the positive influence of a tolerant rootstock on scion antioxidant system under combined stress conditions and the opposite situation (the negative effect of a sensitive rootstock). This work also provides insights on the physiological responses of citrus (as a model tree crop) to guide future agronomical actuations and genetic improvement programs to cope with environmental changes that are probably occurring in the near future due to climate change.

## Materials and methods

### Plant material

**Fig. 1:**
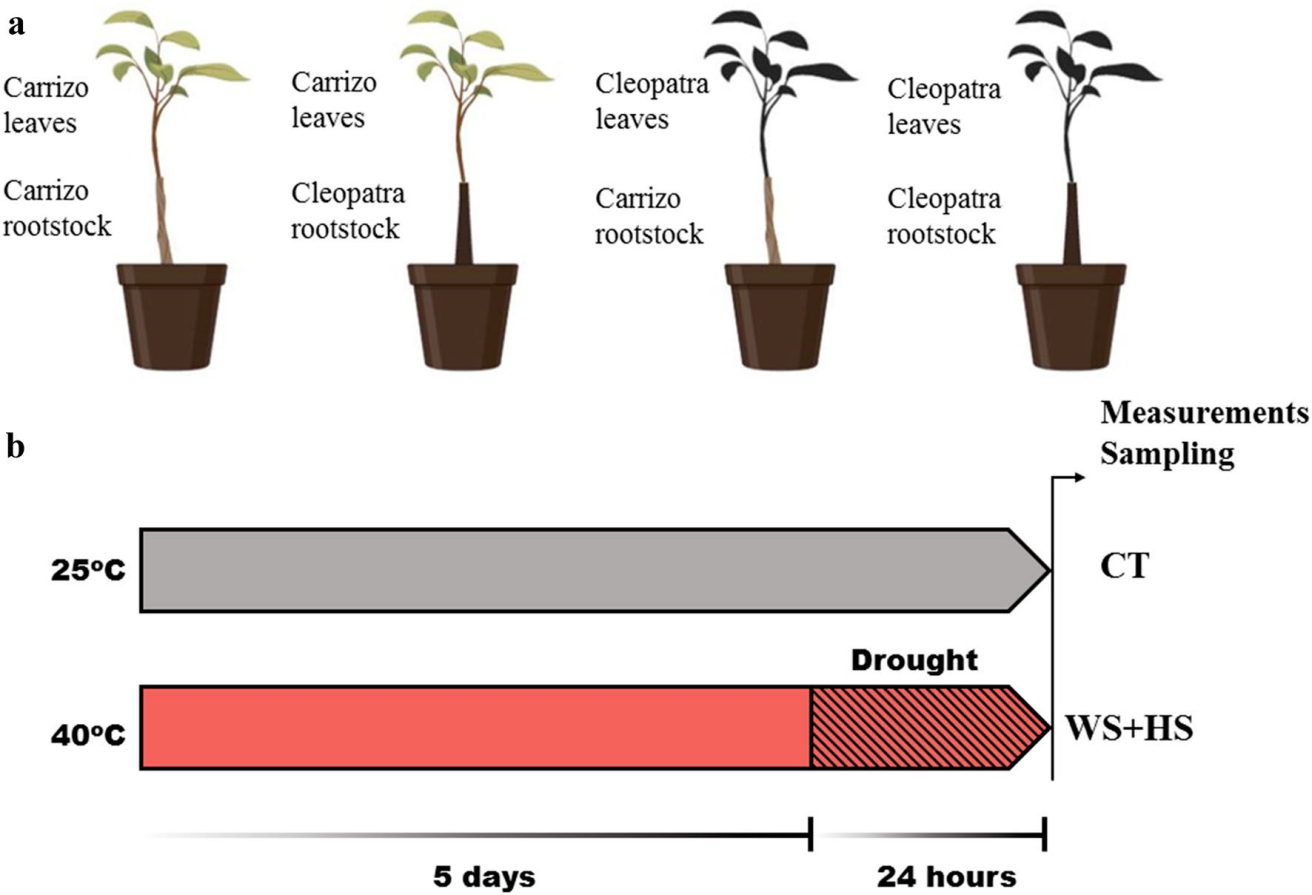
Carrizo citrange and Cleopatra mandarin self- and reciprocal grafts (**a**). Experimental design to study the effect of drought and heat stress combination (WS + HS) in citrus grafted plants (**b**). Images created with BioRender.com

Reciprocal and self-grafted plants of Carrizo citrange (*Poncirus trifoliate* × *Citrus sinensis*) and Cleopatra mandarin (*Citrus reshni*) were purchased from a commercial nursery (Beniplant S.L., Penyíscola, Spain). 2-year-old seedlings of four types of citrus grafting combinations (Fig. 1) were grown in 2.4 L plastic pots filled with perlite and watered three times a week with a half-strength Hoagland solution under greenhouse conditions, with natural photoperiod and day and night temperature averaging 25.0 ± 3.0 °C and 18.0 ± 3.0 °C, respectively. Then, plants were maintained for 2 weeks in growth chambers to acclimate to a 16 h photoperiod at 25 °C and relative moisture at approximately 80%.

### Stress treatments

Similar to Zandalinas et al. (2017), two different groups of treatment were established: well-watered plants at 25 °C (CT) and plants subjected to water stress at 40 °C (WS + HS). Temperature treatment was applied for 6 days whereas water stress was imposed by transferring plants to dry perlite for the last 24 h (Fig. 1).

### Leaf damage and relative water content

Leaf damage was evaluated after stress treatments. Chlorotic or wilted leaves were considered damaged leaves. Relative water content (RWC) of leaves was calculated after stress treatments by using at least three leaves from the same age and position from three different plants, which were weighed to obtain a leaf fresh weight (M_f_). Leaves were allowed to rehydrate overnight in an opaque beaker filled with water. Therefore, they were reweighed to obtain turgid weight (M_t_). Finally, leaves were dried at 80 °C for 48 h to obtain dry weight (M_d_). RWC was calculated as [(M_f_−M_d_) × (M_t_−M_d_)^−1^] × 100 according to Morgan (1984).

### Maximum efficiency of photosystem II (Φ_PSII_) and photosynthesis rate

Φ_PSII_ was measured with a portable fluorometer (FluorPen FP-MAX 100, Photon Systems Instruments, Czech Republic) after stress treatments. Photosynthetic measurements were taken from at least 15 plants using three adult leaves per plant for each stress treatment, and each experiment was repeated at least three times. Photosynthesis rate (A) was measured with a LCpro + portable infrared gas analyzer (ADC BioScientific Ltd., Hoddesdon, UK) under ambient CO_2_ and moisture. Supplemental light was provided by a photosynthetic active radiation (PAR) lamp at 1000 μmol m^−2^ s^−1^ and air flow was set at 150 μmol mol^−1^. After instrument stabilization, at least 10 measurements were taken on three leaves in three replicate plants from each stress treatment.

### H_2_O_2_ and antioxidant enzyme activity

H_2_O_2_ accumulation in leaves was measured by using a commercial kit (Amplex Red hydrogen peroxide-peroxidase assay, Molecular Probes/Invitrogen, Eugene, OR, USA) as described in Balfagón et al. (2019) with few modifications. Briefly, 500 μL of 50 mM sodium P buffer at pH 7.4, containing 50 μM of Amplex Red reagent and 0.05 U mL^−1^ of horseradish peroxidase, was added to approximately 40 mg of leaf ground frozen tissue and incubated for 30 min at room temperature in the dark. Then, samples were centrifuged at 12,000 g for 12 min at 4 °C and 50 μL of supernatant transferred into new opaque tubes. Absorbance at 560 nm was measured by using a NanoDrop Spectrophotometer (Thermo Fisher Scientific, Wilmington, DE, USA). The concentration of H_2_O_2_ in each sample was determined from a standard curve consisting of 0, 0.5, 1, 3, 6, and 9 μM of H_2_O_2_. After absorbance measurement, H_2_O_2_ accumulation per gram of fresh weight was calculated.

For enzymatic activities of APX, SOD and CAT, about 100 mg of frozen ground leaf tissue were extracted in 2 mL of phosphate buffer in a ball mill (MillMix20, Domel, Železniki, Slovenija). After centrifugation at 14000 g for 10 min at 4 °C, supernatant was recovered. Different buffers were used for enzyme extractions as follows: for APX, 50 mM phosphate buffer (pH 7.8) supplemented with 1 mM sodium ascorbate and 1 mM Ethylenediaminetetraacetic acid (EDTA); for SOD, 50 mM phosphate buffer (pH 6.8) with 1.33 mM diethyl-diamino-pentaacetic acid; finally, CAT was extracted in 50 mM phosphate buffer (pH 6.8). The APX activity was assayed following the depletion in absorbance at 290 nm due to ascorbate (AsA) consumption. The SOD activity was determined following the O.^**−**2^-induced reduction of nitroblue tetrazolium using the xanthine–xanthine oxidase system. CAT was determined using the hydrogen peroxide-dependent reduction of titanium chloride. Soluble protein content was determined according to Bradford (1976) using BSA as a standard. Enzyme activity was expressed as U mg^−1^ protein. Further details on enzyme assays are provided in Hossain et al. (2009).

### RT-qPCR gene expression analyses

The specific primers used for the amplification of each gene are included in Supplementary Table S1. RT-qPCR analyses were performed in a StepOne Real-Time PCR system (Applied Biosystems, Foster City, CA, United States). The reaction mixture contained 1 μL of cDNA, 5 μL of SYBRGreen (Applied Biosystems) and 1 μM of each gene-specific primer pair in a final volume of 10 μL. The thermal profile used to analyze the relative gene expression consisted of 10 min at 95 °C for pre-incubation, followed by 40 cycles of 10 s at 95 °C for denaturation, 10 s at 60 °C for annealing and 20 s at 72 °C for the extension. Amplicon specificity of the PCR reaction was evaluated by the presence of a single peak in the dissociation curve after the amplification steps. The expression levels of all genes were normalized against the expression of two endogenous control genes (tubulin and actin) based on previous housekeeping selection for citrus tissues (Mafra et al. 2012) and the relative expression were calculated by using REST (Pfaffl et al. 2002). For all genes studied, the reference sample was the expression value obtained at the non-stressed samples and set at one.

### Protein extraction and western blot analysis

Protein extraction and western blot analyses were carried out as described in Balfagón et al. (2018). Actin blot (β-Actin; 1:5000; product number A3854; Sigma, St. Louis, MO, USA) was used as protein loading controls. Primary antibodies used were: APX2 (orange1.1g024615m, L-Ascorbate peroxidase 2, cytosolic) (product number AS06 180) (1:10,000), Cu/ZnSOD (orange1.1g031837m, Superoxide dismutase 1, Cu–Zn family) (product number AS18 4243) (1:1500) and CAT (orange1.1g042356m, Catalase peroxidase) (product number AS09 501) (1:1000) from Agrisera (Vännäs, Sweden). BlastP analysis were performed between the proteins sequences in *Citrus sinensis* and *Arabidopsis thaliana* with the following results: APX2 (Qc 99%; *E* value 1 e–153; Identity 78%), Cu/ZnSOD (Qc 99%; *E* value 1e–93; Identity 84%), CAT (Qc 99%; *E* value 0.0; identity 87%).

### Statistical analysis

Statistical differences between stress treatments and grafting groups were discriminated by two-way ANOVA followed by a Tukey post hoc test (*p* < 0.05) when a significant difference was detected. For gene expression and protein content quantification, statistical analyses were performed between treatments (CT and WS + HS) of each grafting group, by means of the two-tailed Student’s *t* test.

## Results

### Plant status and physiology

**Fig. 2:**
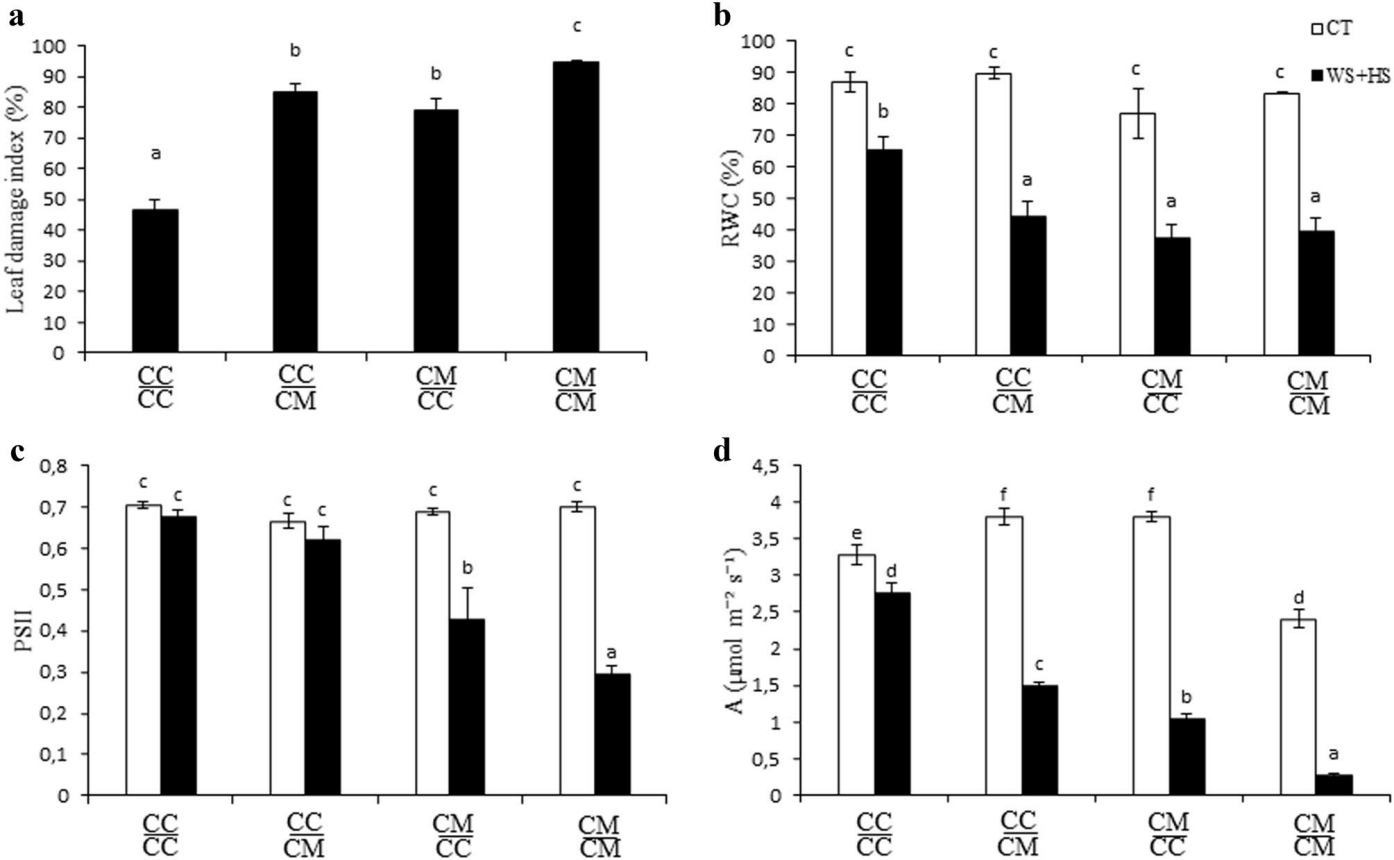
Leaf damage index (**a**), leaf relative water content (**b**), Φ_PSII_ (*n* = 15) (**c**) and photosynthetic rate (*n* = 10) (**d**) of Carrizo and Cleopatra plants self-grafted or grafted into each other after drought and heat stress combination. Data are mean values ± standard errors. Different letters show statistical significance at *p* ≤ 0.05

**Fig. 3:**
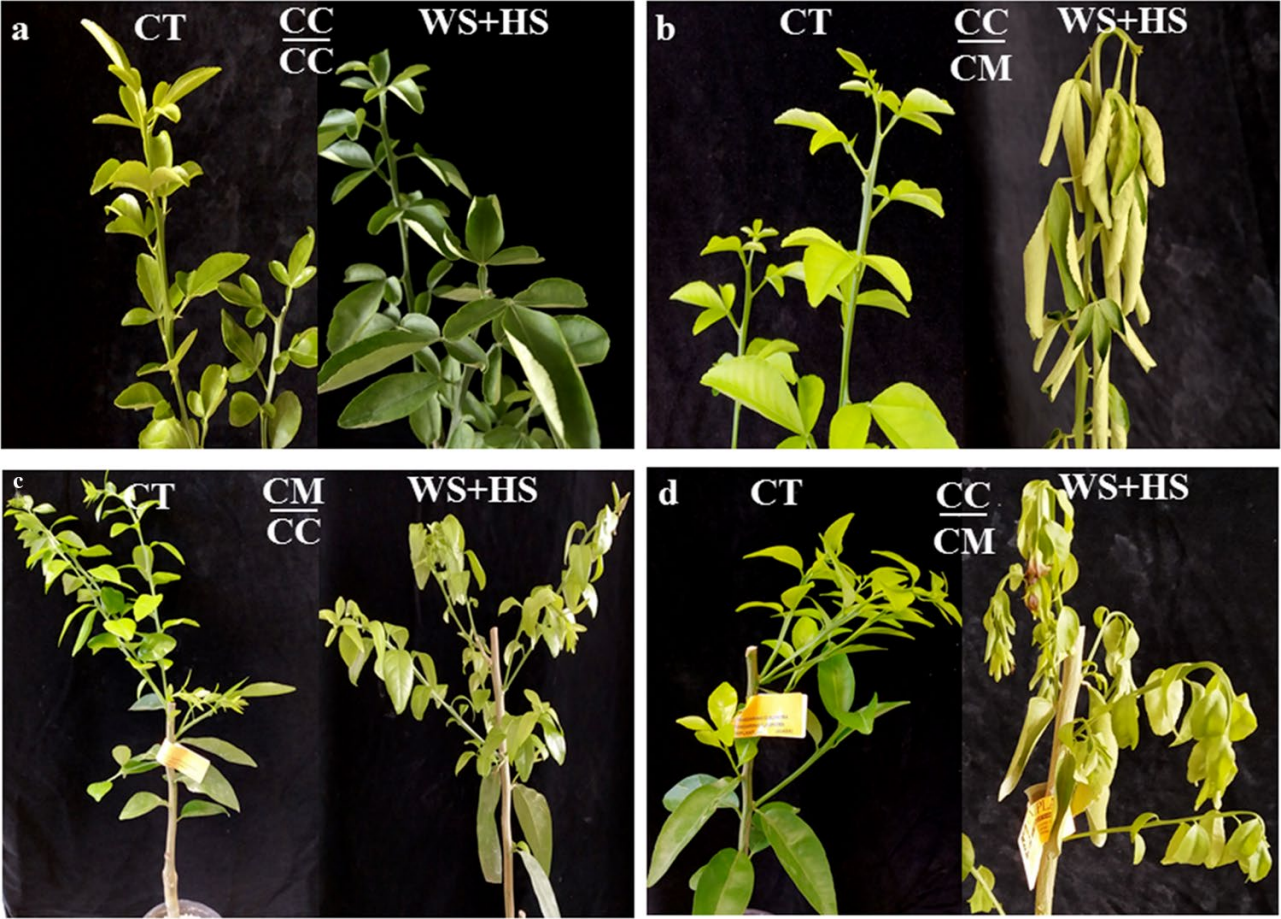
Representative images of Carrizo plants grafted onto Carrizo (**a**) or Cleopatra (**b**) and Cleopatra plants grafted onto Carrizo (**c**) or Cleopatra (**d**), subjected to control or drought and heat stress combination conditions

To study the effect of rootstock on scion tolerance to drought and heat stress combination, citrus grafted plants were subjected to water deficit and high temperatures (Fig. 1, WS + HS). Visible damage was evaluated in plants after stress treatments (Figs. 2a and 3). Stress combination causes extensive leaf damage on all groups of plants. Cleopatra self-grafted $\frac{{{\text{CM}}}}{{{\text{CM}}}}$ plants were the most damaged (94.3% of leaf damage), Carrizo grafted onto Cleopatra $\frac{{{\text{CC}}}}{{{\text{CM}}}}$ and Cleopatra grafted onto Carrizo $\frac{{{\text{CM}}}}{{{\text{CC}}}}$ groups showed significantly less damaged leaves (84.7% and 78.7%, respectively), and Carrizo self-grafted $\frac{{{\text{CC}}}}{{{\text{CC}}}}$ plants were the least affected by stress combination (46.3% of leaf damage). Leaf RWC significantly decreased after WS + HS treatments in all groups of plants, compared to CT values (Fig. 2b). Although, the reduction in RWC of $\frac{{{\text{CC}}}}{{{\text{CC}}}}$ leaves was lower than in the rest of groups. Maximum efficiency of photosystem II (Φ_PSII_) of Cleopatra scions decreased after stress combination, independently of the genotype they were grafted onto, but the decline was lower in $\frac{{{\text{CC}}}}{{{\text{CC}}}}$ plants (Fig. 2c). In Carrizo leaves, no variation of Φ_PSII_ values was observed between controls and stressed plants, despite the rootstock used. Finally, photosynthesis rate (A) of $\frac{{{\text{CM}}}}{{{\text{CM}}}}$ severely decreased after stress combination in comparison to control. Grafting Cleopatra onto Carrizo had a positive effect on A, and the values were higher than in the self-grafted plants. On the other hand, A of $\frac{{{\text{CC}}}}{{{\text{CC}}}}$ plants only slightly decreased in response to the adverse conditions, but this good performance of the photosynthesis rate was impaired when Cleopatra was used as a rootstock (Fig. 2d).

Drought and heat stress combination affected severely to plant performance in both genotypes, as appreciated for a high leaf damage, and reductions in RWC, Φ_PSII_ and A. However, self-grafted plants of Carrizo were less affected than those of self-grated Cleopatra. Interestingly, reciprocal grafted plants showed intermediated tolerances.

### H_2_O_2_ content and antioxidant enzymatic activity

**Fig. 4:**
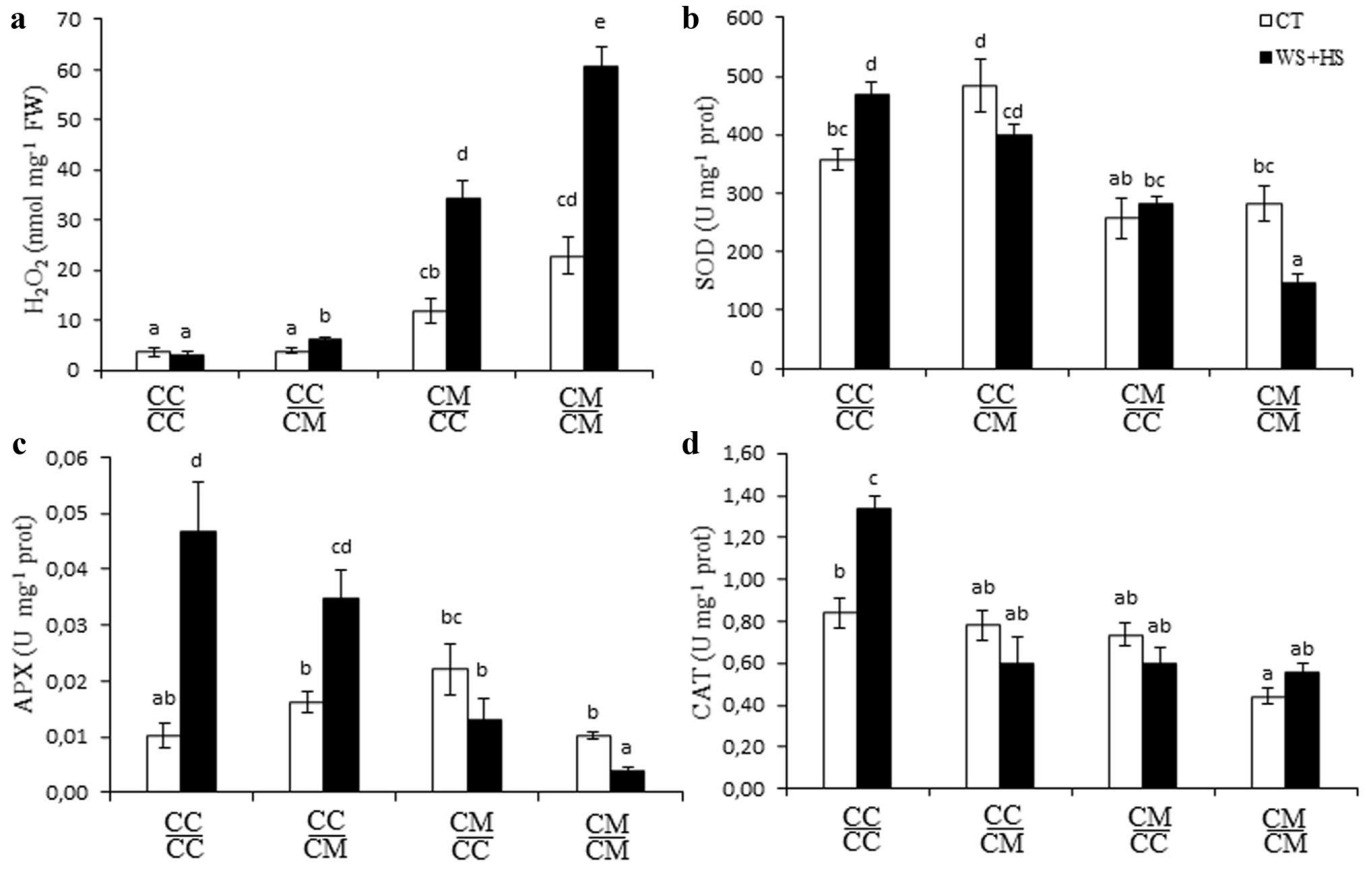
H_2_O_2_ leaf content (**a**) and enzymatic activity of SOD (**b**), APX (**c**) and CAT (**d**) of Carrizo and Cleopatra plants self-grafted or grafted into each other after drought and heat stress combination. Data are mean values ± standard errors (*n* = 3). Different letters show statistical significance at *p* ≤ 0.05

H_2_O_2_ content highly increased in leaves of self-grafted Cleopatra plants after stress combination. The use of Carrizo as a rootstock had a positive effect and the concentration of H_2_O_2_ content in stressed $\frac{{{\text{CM}}}}{{{\text{CC}}}}$ plants was lower than $\frac{{{\text{CM}}}}{{{\text{CM}}}}$ ones. After stress combination, H_2_O_2_ content in Carrizo leaves differed depending on the rootstock, $\frac{{{\text{CC}}}}{{{\text{CC}}}}$ plants did not accumulate more H_2_O_2_ than control plants but $\frac{{{\text{CC}}}}{{{\text{CM}}}}$ plants had a slight increase in this parameter (Fig. 4a). Activity of the antioxidant enzymes SOD, APX and CAT was determined crucial in citrus plants to tolerate drought and heat stress combination (Zandalinas et al. 2017). In this work, we tested the activity of these three enzymes to evaluate the effect of the rootstock on scion antioxidant system under stress combination. SOD activity increased in $\frac{{{\text{CC}}}}{{{\text{CC}}}}$ plants under stress combination in comparison to control. On the contrary, $\frac{{{\text{CM}}}}{{{\text{CM}}}}$ plants under the abiotic stress imposed showed a decline in SOD activity, compared to control. Carrizo and Cleopatra plants grafted onto each other $\frac{{{\text{CC}}}}{{{\text{CM}}}}$ and $\frac{{{\text{CM}}}}{{{\text{CC}}}}$ showed no difference in SOD activity between control and stressed plants (Fig. 4b). Leaf APX activity of Carrizo plants, self-grafted or grafted onto Cleopatra, increased significantly under stress combination, compared to control. In $\frac{{{\text{CM}}}}{{{\text{CM}}}}$ plants, APX activity decreased significantly under drought and heat stress combination in comparison to control values, whereas in $\frac{{{\text{CM}}}}{{{\text{CC}}}}$ plants APX activity did not vary because of the adverse condition (Fig. 4c). Finally, CAT activity in $\frac{{{\text{CC}}}}{{{\text{CC}}}}$ plants increased under stress conditions compared to controls. However, in $\frac{{{\text{CC}}}}{{{\text{CM}}}}$, $\frac{{{\text{CM}}}}{{{\text{CC}}}}$ and $\frac{{{\text{CM}}}}{{{\text{CM}}}}$ plants, CAT activity was not modified by the stress combination (Fig. 4d).

These results indicate that Cleopatra is unable to activate SOD, APX and CAT activity under stress combination, what could be the reason for the increase in H_2_O_2_ leaf content. In addition, Cleopatra as a rootstock modified the enzymatic response of Carrizo scions. Similarly, Carrizo rootstock positively affected Cleopatra aerial part by avoiding a decrease of the antioxidant enzymatic activity under stress.

### Gene expression and accumulation of antioxidant enzymes

**Fig. 5:**
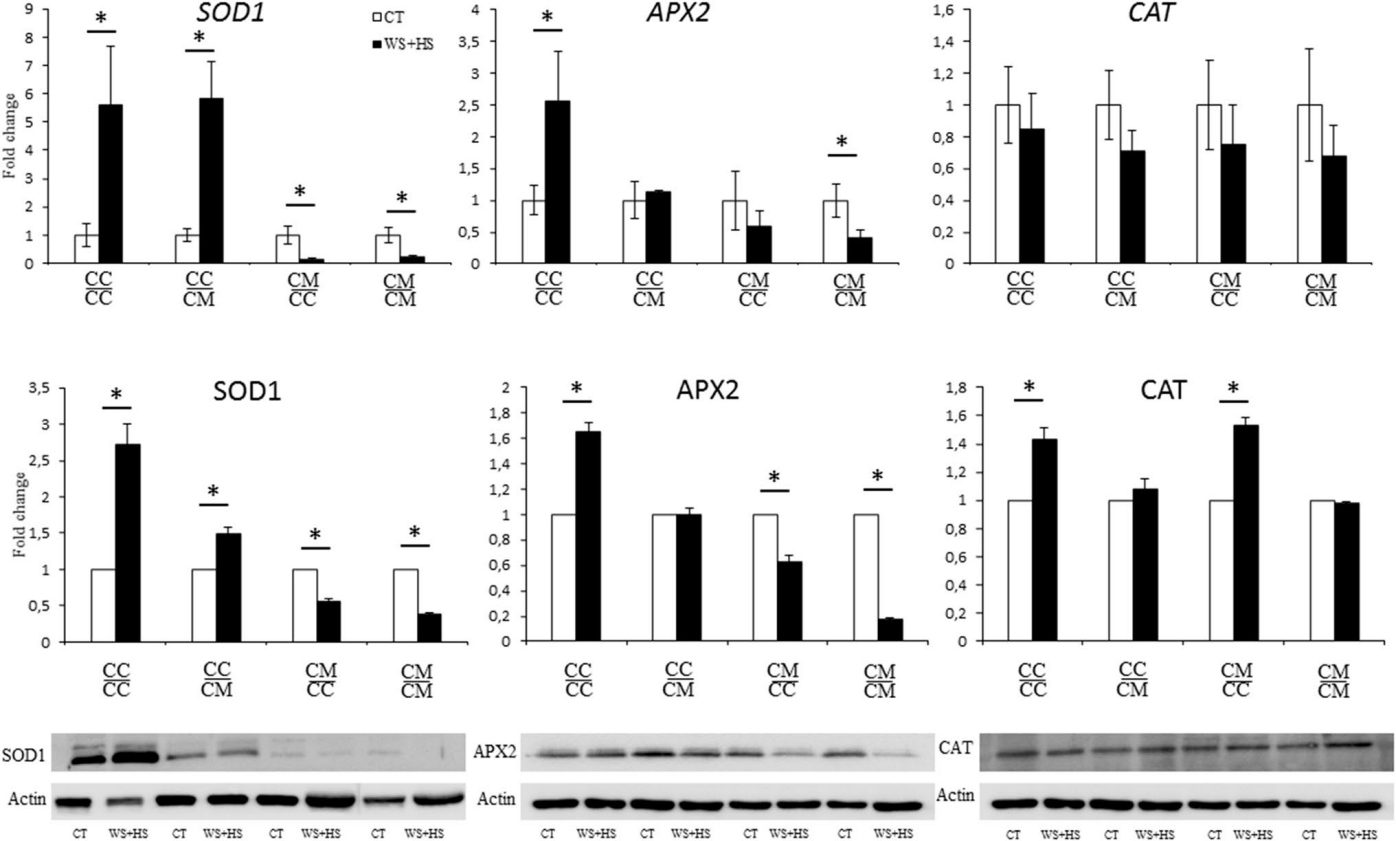
Relative gene expression of SOD1, APX2 and CAT genes (Top) and protein blots and quantitative bar graphs (Bottom) of SOD1, APX2 and CAT proteins. Both analyses were performed in Carrizo and Cleopatra plants self-grafted or grafted into each other after drought and heat stress combination. Data are mean values ± standard errors (*n* = 3). Asterisks denote Student’s *t* test significant at *p* < 0.05 between control and stressed plants within each grafting group

To further analyze the effect of the rootstock on scion antioxidant system under stress combination, RT-qPCR analyses and western blots of SOD1, APX2 and CAT genes and proteins were performed (Fig. 5). Under drought and heat stress combination, a significant increase of gene expression and protein accumulation of SOD1 were observed in $\frac{{{\text{CC}}}}{{{\text{CC}}}}$ and $\frac{{{\text{CC}}}}{{{\text{CM}}}}$ plants, compared to controls. On the contrary SOD1 gene expression and protein content decreased in $\frac{{{\text{CM}}}}{{{\text{CC}}}}$ and $\frac{{{\text{CM}}}}{{{\text{CM}}}}$ plants under stress combination. APX2 gene expression and protein content of Carrizo self-grafted plants increased significantly after stress treatment, whereas in $\frac{{{\text{CC}}}}{{{\text{CM}}}}$ plants levels remained as in control conditions. After stress treatment, APX2 gene expression and protein content decreased significantly in Cleopatra self-grafted plants, whereas in $\frac{{{\text{CM}}}}{{{\text{CC}}}}$ plants gene expression was similar to control and the decrease of protein content was lower (Fig. 5). Finally, under stress, CAT gene expression did not change in any type of plant. However, protein content increased in plants of both genotypes grafted onto Carrizo ($\frac{{{\text{CC}}}}{{{\text{CC}}}}$ and$\frac{{{\text{CM}}}}{{{\text{CC}}}}$) and remained as in controls in plants grafted onto Cleopatra ($\frac{{{\text{CC}}}}{{{\text{CM}}}}$ and$\frac{{{\text{CM}}}}{{{\text{CM}}}}$).

These results show that rootstock directly influenced gene expression and protein content of SOD1, APX2 and CAT in Carrizo and Cleopatra plants under stress combination.

## Discussion

The combination of water deficit and heat stress is an increasingly common situation in the citrus cultivated areas due to climate change. Key physiological and biochemical traits of citrus rootstocks that confers plant tolerance to this stress combination have been identified (Zandalinas et al. 2016, 2017; Balfagón et al. 2018). The use of grating on citrus industry is widely extended as it is known that the rootstock modifies physiological, biochemical and genetic performance of the scion (Clearwater et al. 2007; Marguerit et al. 2012; Martínez-Andújar et al. 2016; Cochetel et al. 2018). Grafting a productive variety onto an adequate rootstock has numerous benefits: shortening of the juvenile period, productivity increase and/or tolerance to abiotic and biotic stresses (Cimen and Yesiloglu 2016; Bowman and Joubert 2020). In Zandalinas et al. (2017), we demonstrated that Carrizo citrange is more tolerant to water deficit and heat stress combination than Cleopatra mandarin due to a higher activation of the antioxidant system. Although tolerance of the rootstock to this stress combination is agronomically important, it is essential to know if this acclimation response is transferred to the scion, since this would be decisive for a proper performance of the canopy and, subsequently, for a sustainable yield. Reciprocal grafting between plants of Carrizo citrange and Cleopatra mandarin, two genotypes with contrasted tolerance to drought and heat stress combination (Zandalinas et al. 2016, 2017; Balfagón et al. 2018), were used to address this question.

In our results, drought and heat combination was a severe adverse condition for plants of both citrus genotypes (Fig. 3). However, under stress, Cleopatra self-grafted plants had more damaged leaves, less RWC and lower Φ_PSII_ and photosynthetic rate than those of Carrizo self-grafted (Fig. 2). These results are in accordance with those reported in Zandalinas et al. (2016), where Carrizo intact plants were less damaged and photosynthesis rate decline was lower than in Cleopatra ones. In this work, we are further demonstrating that physiology under stress combination in both genotypes was modified when grafted onto a tolerant or sensitive rootstock. Focusing on the scion, we observed that Carrizo plants lost part of their ability to cope with drought and heat stress combination when grafted onto Cleopatra. On the contrary, with Carrizo as rootstock, Cleopatra scion improved its resilience to this condition (Fig. 2). Previous studies demonstrated that drought and heat stress combination caused a reduction of photosynthetic activity in Arabidopsis and tobacco (Rizhsky et al. 2002, 2004). In *Populus yunnanensis*, this stress combination led to a reduction of photosynthesis rate together with an overaccumulation of ROS (Li et al. 2014). In addition, Zandalinas et al. (2016) suggested that in citrus plants under stress combination, net photosynthesis ratio was reduced because of stomatal closure signaling, in part by ROS, and the impairment of PSII. In our results, the sensitive genotype, Cleopatra, had higher levels of H_2_O_2_ under stress combination what likely caused more PSII damage and a strong photosynthesis rate reduction. However, scion physiology performance improved when Cleopatra was grafted onto tolerant Carrizo plants and, all the parameters measured indicated that the grafted plants were less affected by the stress combination (Fig. 2). On the contrary, significant accumulation of H_2_O_2_ and, consequently, strong decline of photosynthesis in Carrizo scion under stress combination were induced by grafting them onto the sensitive rootstock, Cleopatra. Photosynthesis can be highly affected by ROS inhibition of PSII repairment, since PSII is the most sensitive component of the photosynthetic apparatus to drought and, specially, to high temperatures (Mathur et al. 2014; Chen et al. 2016).

Metabolic alterations cause imbalances in ROS production and detoxification, what leads to overaccumulation that exposes cells to high levels of oxidative damage (Suzuki et al. 2012). The increase of activity and content of the antioxidant enzymes SOD, APX and CAT, involve in ROS detoxification, is a key response to acclimation to single or multiple abiotic stresses (Mittler 2002; Hossain et al. 2009; Choudhury et al. 2017; Zandalinas et al. 2017; Balfagón et al. 2018). In the present study, expression of APX2 together with the content and activity of SOD, CAT and APX were altered in scions depending on the rootstock they were grafted onto (Figs. 4 and 5). Under drought and heat stress combination, Carrizo showed higher antioxidant enzymatic activity than Cleopatra (see also Zandalinas et al. 2017). Furthermore, activation of the antioxidant defense system to cope with oxidative stress seems to be a transmissible trait from the rootstock to the scion as indicated by the fact that grafting Cleopatra onto Carrizo increased the antioxidant activity and the total content of antioxidant enzymes of the scion (Figs. 4 and 5). In this case, the expression of genes coding for the different enzymes was not altered. This transmissibility is further supported by the opposite situation as the scion can be also negatively affected by a sensitive rootstock when dealing with excessive ROS derived from stress combination. Therefore, under stress, CAT activity and protein content of the three enzymes were reduced in Carrizo plants grafted onto Cleopatra when compared with self-grafted plants (Figs. 4 and 5).

SOD, APX and CAT activities in citrus scions were modified by the rootstock under stress combination, except for CAT activity on $\frac{{{\text{CM}}}}{{{\text{CC}}}}$ plants (Fig. 4). However, not always enzymatic activity variations correlated with protein content of SOD1, APX2 and CAT (Fig. 5). For example, stress combination induced SOD1 accumulation in $\frac{{{\text{CC}}}}{{{\text{CM}}}}$ plants respect to control whereas SOD activity remained as control values in those plants (Fig. 4b). The lack of correlation between enzymatic activity and the total content of studied proteins can be explained by the different types of SOD and APX isoforms that exist in the plant cells. All these forms can contribute to total antioxidant activity. However, in this work, blot analyses were performed for the specific proteins: Cu/ZnSOD and cytosolic APX (Mittler 2002; Asada 2006; Foyer and Shigeoka 2011). In addition, SOD, APX and CAT are metalloenzymes that depend on metallic cofactors, that may be available or not, to perform their catabolic processes (Anjum et al. 2016; Sachdev et al. 2021). Therefore, the increase in protein content does not always correlates with a higher enzymatic activity. Similarly, gene expression did not always correlate with protein accumulation (e.g., for CAT), and this can be justified by post-transcriptional regulations that finally determine protein homeostasis. Nonetheless, the blot analyses show that there is a direct influence of the rootstock on the content of leaf antioxidant enzymes when plants were subjected to stress combination and contents of the three studied proteins were modified by the presence of tolerant or sensitive rootstocks (Fig. 5).

When grafting two plants, their vascular systems, xylem and phloem, are brought into contact. This allows the trafficking of transmissible molecules between the rootstock and the scion that are capable of acting at a distance to influence physiological processes or trigger responses to environmental cues. Only a few molecules that move through the vascular conduits have been demonstrated to be signaling molecules, which include hormones, proteins, small peptides, mRNAs or small RNAs (Haroldsen et al. 2012; Turnbull and Lopez-Cobollo 2013; Thomas and Frank 2019). In response to environmental stresses, molecules produced in the rootstock can move to the scion to trigger tolerance responses, such as activation of the antioxidant activity to cope with oxidative stress (López-Gómez et al. 2007; He et al. 2009; Li et al. 2016). Moya et al. (2002) also demonstrated that some morphologic traits such as water uptake reduction and lower leaf Cl^−^ accumulation are transmissible in citrus under salt stress. In the present study, we have demonstrated the ability of the rootstock to transfer traits for stress combination tolerance to the scion. Thus, Carrizo citrange seems a better rootstock than Cleopatra mandarin under drought and heat stress combination because it is capable to transfer its efficient antioxidant response to the scion alleviating leaf and photosynthetic damage derived from oxidative stress. Finally, these results shed light on the key traits to choose rootstocks that can sustain citrus yield under the threatening environmental conditions that can often concur in the fields due to climate change.

## Supplementary Information

Below is the link to the electronic supplementary material.

Supplementary file1 (DOCX 14 KB)
