# Supplementary material for: Citrus rootstocks modify scion antioxidant system under drought and heat stress combination
Source: Plant Cell Rep. 2021 Jul 7;41(3):593–602. doi: 10.1007/s00299-021-02744-y (PMC8989854; doi:10.1007/s00299-021-02744-y)
Supplement: Supplementary file 1 — Supplementary file1 (DOCX 14 KB) [file 299_2021_2744_MOESM1_ESM.docx]

| **Citrus gene** | **Locus** | **Forward /**  **Reverse** | **Sequence (5’🡪3’)** | **Amplicon size (bp)** |
| --- | --- | --- | --- | --- |
| **Cu/Zn SOD** | orange1.1g031837m | F | CTTGGTGGAACTGAGGGTGT | 173 |
|  |  | R | GGGTTAAAGTGGGGTCCAGT |  |
| **CAT** | orange1.1g042356m | F | GTAACCAAGACCTGGCCTGA | 134 |
|  |  | R | ATGCCAGGAACCACAATAGC |  |
| **APX** | orange1.1g024615m | F  R | CCATTCGGAACCATGAGGCT CTCAACGCCAACAACACCAG | 153 |
| **ACT** | orange1.1g037845m | F | CCCTTCCTCATGCCATTCTTC | 105 |
|  |  | R | CGGCTGTGGTGGTAAACATG |  |
| **TUB** | orange1.1g013335m | F | GGGGCAAAATGAGCACTAAA | 187 |
|  |  | R | CGCCTGAACATCTCCTGAAT |  |

**Table S1**. Designed primers for gene expression analyses by quantitative RT-qPCR.
